# Extended therapy with [^177^Lu]Lu-PSMA-617 in responding patients with high-volume metastatic castration-resistant prostate cancer

**DOI:** 10.1007/s00259-023-06119-1

**Published:** 2023-01-27

**Authors:** Nicolai Mader, Christina Nguyen Ngoc, Bilge Kirkgöze, Justus Baumgarten, Daniel Groener, Konrad Klimek, Christian Happel, Nikolaos Tselis, Felix K. H. Chun, Frank Grünwald, Amir Sabet

**Affiliations:** 1 Department of Nuclear Medicine, University Hospital Frankfurt, Frankfurt am Main, Germany; 2 Department of Radiation Oncology, University Hospital Frankfurt, Frankfurt am Main, Germany; 3 Department of Urology, University Hospital Frankfurt, Frankfurt am Main, Germany

**Keywords:** Prostate-specific membrane antigen (PSMA), [^177^Lu]Lu-PSMA, Radioligand therapy (RLT), Extended treatment, Metastatic castration-resistant prostate cancer (mCRPC)

## Abstract

**Purpose:**

The currently used scheme for radioligand therapy (RLT) of patients with metastatic castration-resistant prostate cancer (mCRPC) consists of 4–6 cycles of 6.0–7.4 GBq [^177^Lu]Lu-PSMA-617 each. This standard treatment scheme has proved safe and effective resulting in objective response in most patients with no significant toxicity. Many patients, however, show high-volume residual tumor burden after the sixth cycle and may benefit from treatment continuation. Extended treatment with additional cycles has been withheld due to concerns on potential increased toxicity.

**Methods:**

Twenty-six patients with high-volume residual tumor burden (according to CHAARTED) after standard RLT with [^177^Lu]Lu-PSMA-617 and no alternative treatment option received additional RLT cycles reaching a median of 10 (range 7–16) cycles with a mean activity of 7.4 ± 0.9 GBq per cycle. Response assessment with [^68^Ga]Ga-PSMA-11 PET/CT was done every 2–3 cycles or if disease progression was clinically suspected or based on change in PSA value (according to the PCWG3 criteria). Toxicity was measured using routine blood work up including blood counts, liver and renal function, and was graded according to CTCAE v5.0 criteria. Survival outcome was calculated based on the Kaplan-Meier method.

**Results:**

Further PSA decline of 33 ± 28% during the extended treatment was observed in 21/26 (81%) patients, whereas 5/26 (19%) patients showed a PSA increase; correspondingly in 11/21 patients with an initial response (PR or SD) to extended cycles, treatment was discontinued due to progressive disease, whereas six (23%) patients achieved low-volume residual disease. Two (8%) patients died without showing progression, and two (8%) patients are still under therapy. The median progression-free survival was 19 (95% CI: 15–23) months, and the overall survival was 29 (95% CI: 18–40) months. Grade ≥ 3 hematological toxicities occurred in 4/26 (15%) patients during treatment extension, and nephrotoxicity (grade ≥ 3) was observed in 1/26 (4%) patient during the follow-up.

**Conclusion:**

Extended radioligand therapy is a feasible treatment option in patients with high-volume residual tumor after the completion of standard treatment with six cycles of [^177^Lu]Lu-PSMA-617. Improved survival and the acceptable safety profile warrant further investigation of the concept of additional cycles in selected patients.

## Background

Several systemic treatment options exist for patients with metastatic castration-resistant prostate cancer (mCRPC), predominantly including taxane-based chemotherapies (e.g., docetaxel and cabazitaxel) and new-generation antiandrogen drugs (e.g., enzalutamide and abiraterone). In more recent years, radioligand therapy (RLT) has been introduced for mCRPC patients ineligible for or refractory to other treatment strategies [1]. For this purpose, a selective radiolabeled ligand is used targeting prostate-specific membrane antigen (PSMA), a transmembrane glycoprotein avidly expressed on the surface of prostate cancer cells [2, 3]. [^177^Lu]lutetium is considered the suitable radioisotope for RLT due to its favorable physical characteristics such as its long half-life and short range of emitted medium-energy beta particles. [^177^Lu]Lu-PSMA-617 is the most widely used compound for PSMA-targeted RLT with promising antitumor activity and low toxicity.

Initial experiences with 1–3 cycles of 6 GBq [^177^Lu]Lu-PSMA-617 ([^177^Lu]Lu-PSMA RLT) showed the therapeutic potential of RLT [4, 5]. Subsequent investigations were performed with up to six cycles, demonstrating encouraging results with low toxicity. The current standard treatment scheme consisting of 4–6 cycles of 6–8 GBq [^177^Lu]Lu-PSMA-617 per cycle proved safe and very effective in the randomized VISION phase III trial, prolonging imaging-based progression [6, 7]. Responders, however, frequently show relevant remaining tumor burden after completion of standard [^177^Lu]Lu-PSMA RLT with six cycles. Exhausting treatment alternatives, these patients may benefit from treatment continuation while being exposed to a tolerable risk of treatment-related hematological or renal adverse events. Few studies included patients receiving more than six cycles [^177^Lu]Lu-PSMA RLT in a re-treatment context, but the safety of continued [^177^Lu]Lu-PSMA RLT with more than six cycles has been addressed in only one patient group aiming for complete response [8–11].

This retrospective study assesses the feasibility of extended RLT in patients showing high-volume disease after six cycles of [^177^Lu]Lu-PSMA-617. Treatment continued until progression, significant impairment of bone marrow or renal function, or achieving low-volume residual disease not necessitating further [^177^Lu]Lu-PSMA RLT cycles.

## Methods

### Patients

We retrospectively analyzed 26 patients with mCRPC who successfully completed standard RLT with six cycles of [^177^Lu]Lu-PSMA-617 and received further treatment cycles due to high residual tumor burden. [^177^Lu]Lu-PSMA RLT was performed on a compassionate use basis under the German Pharmaceutical Act §13 (2b). Treatment initiation and continuation were decided by a multidisciplinary tumor board. Patient characteristics at baseline are outlined in Table 1. All patients had progressive, high-volume PSMA-expressing disease in [^68^Ga]Ga-PSMA-11 PET/CT imaging before the commencement of [^177^Lu]Lu-PSMA RLT and showed objective response to standard [^177^Lu]Lu-PSMA RLT with six cycles. High-volume disease was defined according to the CHAARTED criteria (presence of visceral metastasis or ≥ 4 bone lesions with ≥ 1 beyond the vertebral bodies and pelvis [12]). [^177^Lu]Lu-PSMA RLT was continued in responding patients showing high-volume residual disease (multifocal [*n* = 18] or disseminated/diffuse [*n* = 8] bone involvement; visceral involvement [*n* = 5]) in [^68^Ga]Ga-PSMA-11 PET/CT performed after the sixth cycle. Other prerequisites for extended [^177^Lu]Lu-PSMA RLT were estimated glomerular filtration rate (eGFR) of > 30 mL/min/1.73 m^2^, white blood cells (WBC) ≥ 2.00 × 10^9^/L, platelets (Plt) ≥ 75 × 10^9^/L, and hemoglobin (Hb) ≥ 8.0 g/dL after the first six cycles.

**Table 1:** Patient characteristics at initiation of [^177^Lu]Lu-PSMA RLT

	*All patients (n* = *26)*
Age	72 (68–77)
Gleason score	
4–7	6 (23)
8–10	15 (58)
Unknown	5 (19)
VAS	
≥ 4	9 (35)
< 4	17 (65)
ECOG	
1	13 (50)
2	12 (46)
3	1 (4)
PSA at [^177^Lu]Lu-PSMA RLT initiation (ng/mL)	108 (54.2–698.0)
Alkaline phosphatase (U/L)	194 ± 257
Lactase dehydrogenase (U/L)	267 ± 112
Previous treatment	
Abiraterone	22 (85)
Enzalutamide	19 (73)
[^223^Ra]RaCl_2_	9 (35)
Docetaxel	16 (62)
Cabazitaxel	8 (31)
Sites of involvement	
Bone	26 (100)
Oligofocal	0 (0)
Multifocal	14 (54)
Disseminated	7 (27)
Diffuse	5 (19)
Lymph nodes	17 (65)
Visceral	7 (27)
Primary	6 (23)

Data are presented as median with interquartile range (IQR), mean ± standard deviation, or *n* (%)

*[*^*177*^*Lu]Lu-PSMA RLT* [^177^Lu]Lu-PSMA-617 radioligand therapy, *PSA* prostate-specific antigen, *eGFR* estimated glomerular filtration rate, *VAS* visual analog scale, *ECOG* Eastern Cooperative Oncology Group

Extended treatment cycles were intended until achieving a low-volume residual disease state in [^68^Ga]Ga-PSMA-11 PET/CT. Extended [^177^Lu]Lu-PSMA RLT was discontinued upon progression in [^68^Ga]Ga-PSMA-11 PET/CT, significant renal toxicity, irreversible and refractory hematological toxicity, insufficient PSMA expression (≤ liver uptake) in remaining tumor lesions, or clinical deterioration (Eastern Cooperative Oncology Group (ECOG) performance status > 3). All patients gave their written consent after being thoroughly informed about the risks and side effects of this therapy and consented to publication of their data in accordance with the Declaration of Helsinki. The study was approved by the local Institutional Review Board (ethics committee permission number 310/18).

### Radioligand therapy with [^177^Lu]Lu-PSMA-617

[^177^Lu]Lu-PSMA RLT was performed as inpatient procedure at the nuclear medicine therapy ward every 6–8 weeks with an intended activity of 7.4 GBq per cycle. ABX (Advanced Biochemical Compounds GmbH, Radeberg, Germany) provided the PSMA-617 ligand which was labeled in-house with [^177^Lu]LuCl_3_ (ITM Isotopen Technologien München AG, Garching/Munich, Germany) as described in detail previously [13, 14]. Salivary glands were cooled with ice packages for 2 h beginning 30 min before the administration to support xerostomia limitation. [^177^Lu]Lu-PSMA-617 was administered intravenously in 30–60 s preceded and followed by the infusion of 1000 mL sodium chloride 0.9% solution. Renal dosimetry was conducted using planar whole-body scintigraphy and single-photon emission computerized tomography (SPECT) performed at 24 h, 48 h, and 72 h p.i. Patients’ renal masses were determined using CT images.

### Response and toxicity assessment

PSMA-based imaging response was assessed after every 2–3 cycles or if disease progression was suspected using prostate-specific antigen (PSA) as a tumor marker. The international consensus on [^68^Ga]Ga-PSMA-11 PET/CT imaging response assessment was used for evaluation [15]: partial response (PR, reduction of uptake and tumor PET volume by > 30%), stable disease (SD, uptake and tumor PET volume ±  ≤ 30%; no new lesions), and progression (PD, appearance of > 2 new lesions or uptake or tumor PET volume ≥ 30% increased). PSA progression was defined according to the PCWG3 criteria as ≥ 25% increase exceeding 2 ng/mL, confirmed by a second measurement ≥ 3 weeks apart [16].

Hematological (Hb, WBC, and Plt) and renal (eGFR) toxicity evaluation was performed through a blood workup routine at baseline, prior to each therapy cycle, 2–4 weeks after each cycle, and in 6–12-week intervals throughout follow-up. Severity of adverse events was graded based on Common Terminology Criteria for Adverse Events, version 5.0 (CTCAE v5.0) with grade ≥ 3 toxicities considered significant. Mouth dryness was evaluated at every [^177^Lu]Lu-PSMA RLT cycle using a modified, self-reported eight-item xerostomia questionnaire [17]. The visual analog scale (VAS; ranging from 0 to 10) was assessed at baseline and at each treatment cycle and used to evaluate the pain level. The ECOG scale was used to assess the patients’ performance status.

### Statistical analysis

Statistical analyses were performed using the SPSS software (IBM SPSS Statistics 28.0, Armonk, NY, USA). GraphPad Prism version 9.1.1 (GraphPad Software, San Diego, CA, USA) was used to plot graphs. The significance level was set two-sided at *p* < 0.05. The paired sample *t*-test was used to compare intraindividual changes in continuous biochemical parameters. Imaging-based progression-free survival (PFS) was defined as the time interval from [^177^Lu]Lu-PSMA RLT initiation to the date of the first progression, or death, if no imaging-based progression occurred. Overall survival (OS) was defined as the time from treatment initiation to death from any cause; censoring was done if the patient was alive at the time of analysis. PFS and OS were determined using the Kaplan-Meier method (log-rank testing).

#### RESULTS

### Response and survival

**Fig. 1:**
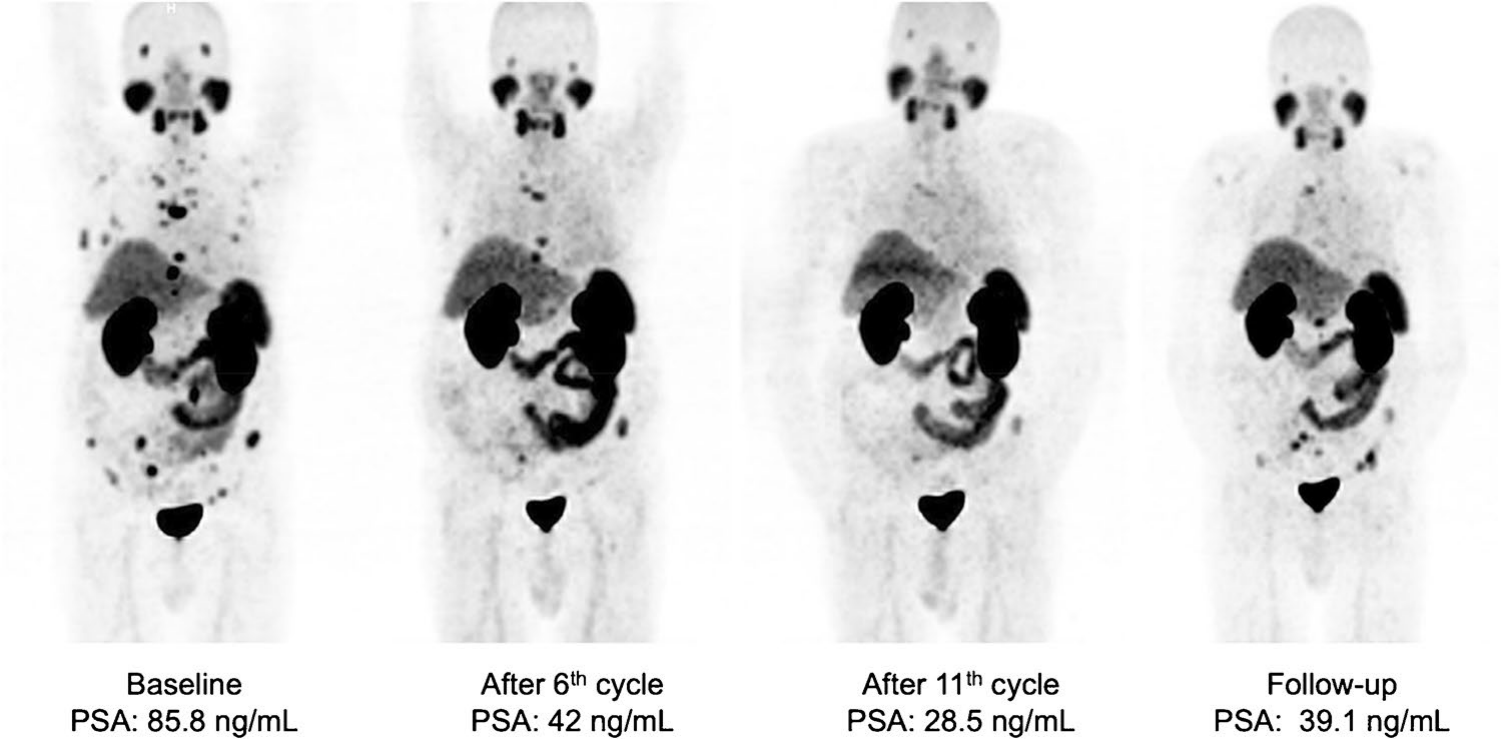
From left to right: maximum-intensity projections (MIP) of [^68^Ga]Ga-PSMA-11 PET images of a patient with multifocal bone and lymph node metastases responding to the standard treatment with six cycles and showing further response to the extended RLT with low-volume residual disease after a total of 11 cycles of [^177^Lu]Lu-PSMA RLT (PFS: 40 months)

**Fig. 2:**
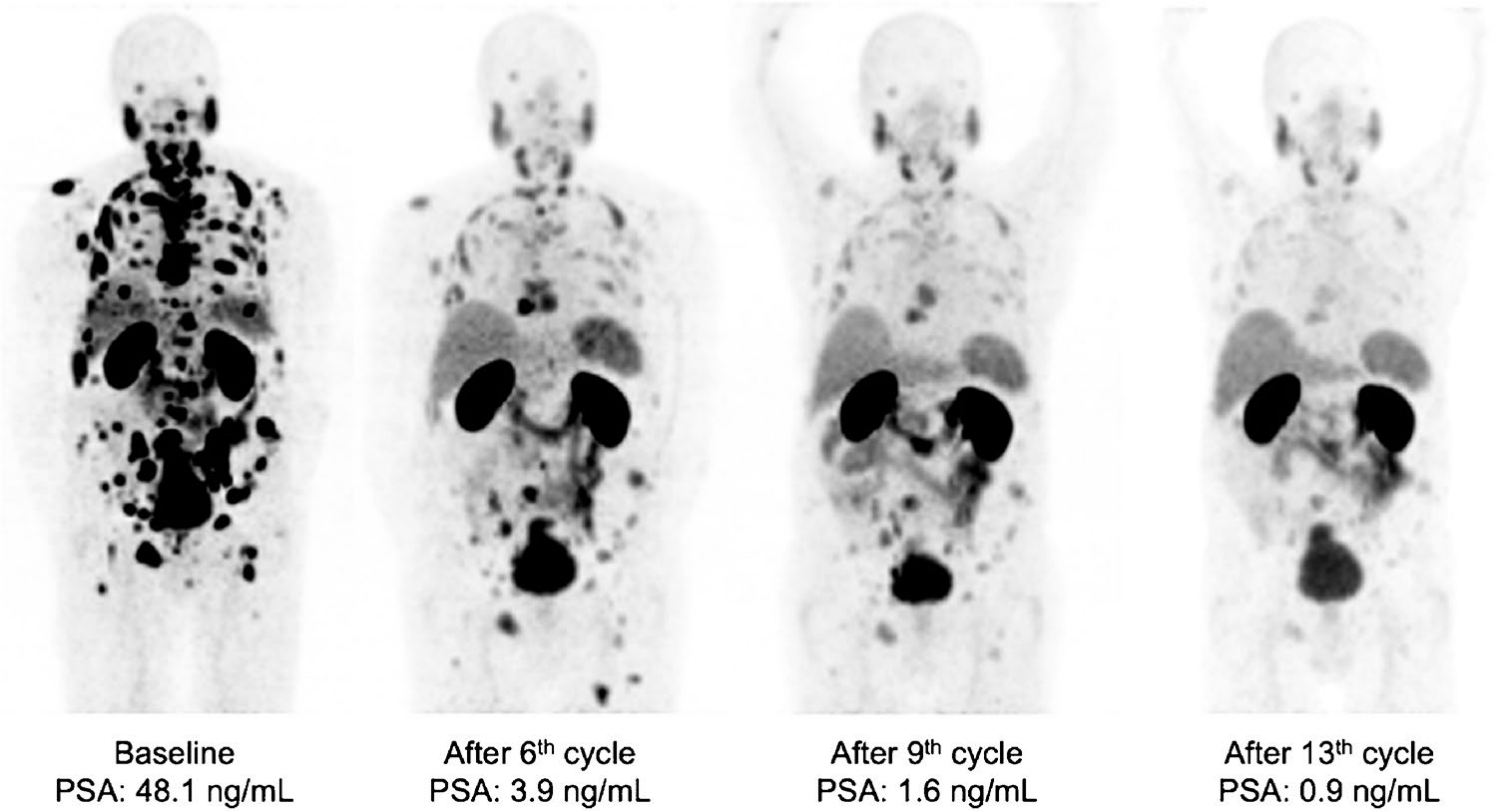
From left to right: MIP of [^68^Ga]Ga-PSMA-11 PET images of a patient with disseminated bone metastases and local disease responding to the standard treatment with six cycles and showing further response to the extended RLT. Treatment was discontinued due to insufficient PSMA-expressing residual disease after 13 cycles of [^177^Lu]Lu-PSMA RLT (PFS: 58 months)

**Fig. 3:**
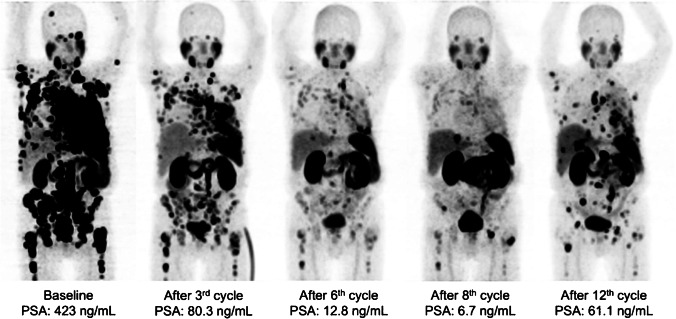
From left to right: MIP [^68^Ga]Ga-PSMA-11 PET images of a patient with disseminated bone and lymph node metastases, pleural carcinosis along with infiltration of the thoracic wall, lymph node metastases, and local disease responding to the initial cycles of extended treatment. [^177^Lu]Lu-PSMA RLT was discontinued upon progression after 12 cycles (PFS: 21 months)

**Fig. 4:**
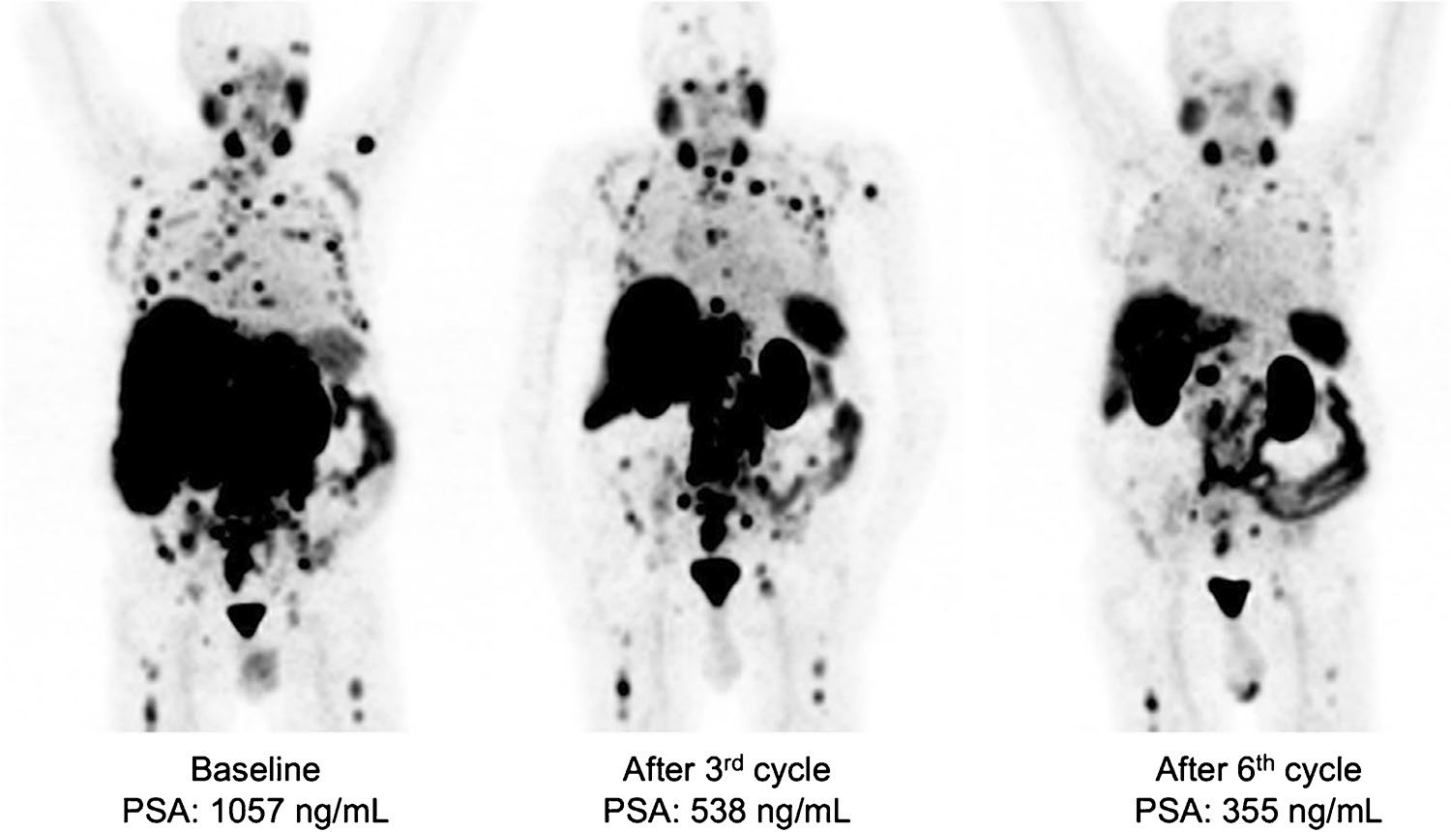
From left to right: MIP [^68^Ga]Ga-PSMA-11 PET images of a responding patient with disseminated lymphatic, skeletal, and hepatic metastatic disease who died after the 7^th^ cycle of [^177^Lu]Lu-PSMA-617 without showing progressive disease

**Fig. 5:**
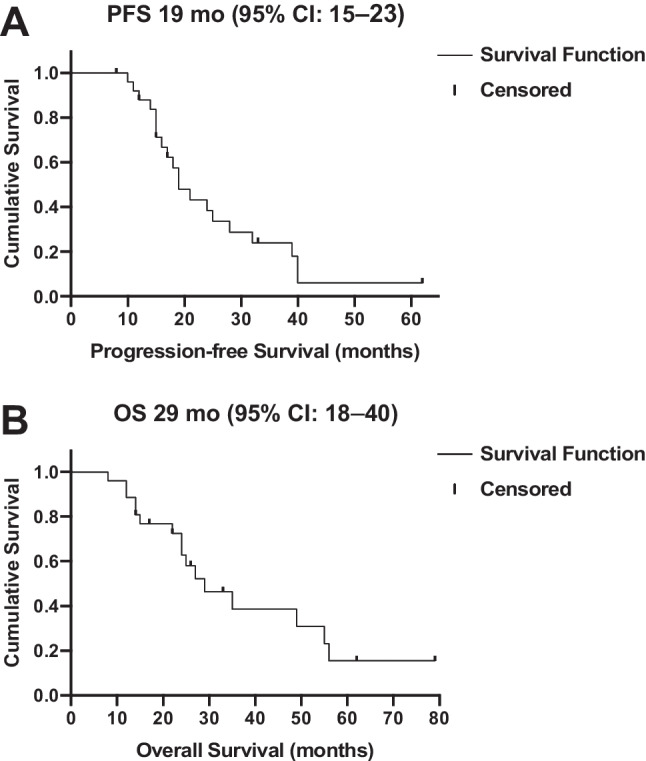
**A** Progression-free survival and **B** overall survival of the patients (*n* = 26) in months

Twenty-six patients who responded to six cycles RLT with [^177^Lu]Lu-PSMA-617 received a median of 4 (IQR 2–6) cycles during extended [^177^Lu]Lu-PSMA RLT reaching 10 (range 7–16) cycles and a cumulative activity of 75.0 ± 19.1 GBq. In 5/26 (19%) patients, PSA increased despite 1 to 2 additional cycles and disease progression was confirmed on [^68^Ga]Ga-PSMA-11 PET/CT imaging, leading to treatment termination. In the remaining 21/26 patients showing objective response in [^68^Ga]Ga-PSMA-11 PET/CT after six cycles, a further PSA decline of 33 ± 28% was observed after initiation of extended treatment, corresponding to an imaging-based partial response (PR) in 10 patients and stable disease (SD) in 11 patients. Six (23%) patients continued treatment until achieving low-volume disease (*n* = 5, Fig. 1) or insufficient PSMA expression in remaining tumor lesions (*n* = 1, Fig. 2). In 11 (42%) patients with initial response (PR + SD) to extended [^177^Lu]Lu-PSMA RLT, progression after subsequent cycles resulted in treatment discontinuation (Fig. 3). Two (8%) patients are still under treatment, and two (8%) patients died without showing disease progression or significant toxicity (Fig. 4). No treatment-induced clinical deterioration or death was recorded. Performance status improved in all nine patients with an uncontrolled pain state (VAS ≥ 4, ECOG 2; Table 1) at baseline as a result of significant pain reduction (> 2 VAS reduction) during [^177^Lu]Lu-PSMA RLT. Altogether, 16/26 (62%) patients died by the time of this analysis. Median PFS was 19 (95% CI: 15–23) months, and median OS was 29 (95% CI: 18–40) months. The Kaplan-Meier survival curves are displayed in Fig. 5.

### Safety

**Fig. 6:**
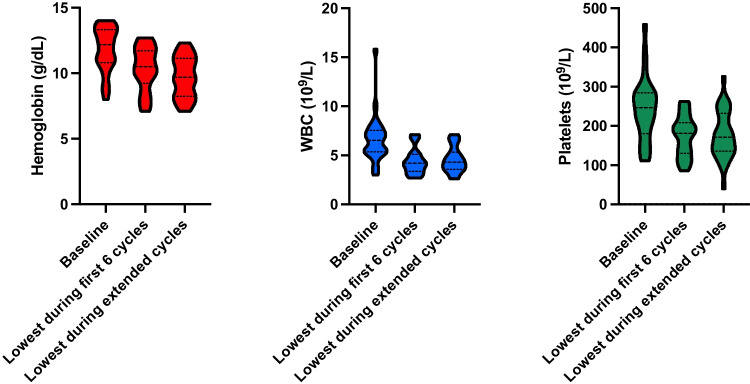
Violin plots for hemoglobin, white blood cells (WBC), and platelets at baseline and upon maximum deterioration during the first six and extended cycles

Hematological parameters showed a significant decline during the extended [^177^Lu]Lu-PSMA RLT (Fig. 6). Mean Hb changed from 11.8 ± 1.7 to 9.7 ± 1.6 g/dL (*p* < 0.001), WBC from 6.5 ± 2.5 to 4.6 ± 1.5 × 10^9^/L (*p* < 0.001), and Plt from 239 ± 82 to 175 ± 65 × 10^9^/L (*p* < 0.001; Table 2). No significant hematotoxicity was observed during the standard [^177^Lu]Lu-PSMA RLT. Four patients (15%), however, developed significant hematological toxicity (grade ≥ 3) in at least one parameter during the extended treatment period: anemia in 4 (15%) patients managed by transfusions and reversible thrombocytopenia in one (4%) patient. Significant leukopenia was not observed.

**Table 2:** Hematological and renal parameters: prior to [^177^Lu]Lu-PSMA RLT, during the standard and extended cycles, and at the last follow-up

Blood parameter	Baseline	Nadir standard		After six cycles	Nadir extended	Last follow-up
Hb (g/dL)	11.8 ± 1.7	10.3 ± 1.7	11.1 ± 1.7		9.7 ± 1.6	9.9 ± 1.7
WBC (10^9^/L)	6.5 ± 2.5	4.4 ± 1.3	5.3 ± 1.6		4.6 ± 1.5	5.1 ± 1.4
Plt (10^9^/L)	239 ± 82	175 ± 52	203 ± 68		175 ± 65	191 ± 75
eGFR (mL/min/1.73m^2^)	85.3 ± 18.9	73.7 ± 18.7	80.6 ± 16.7		67.9 ± 18.1	65.8 ± 19

**Fig. 7:**
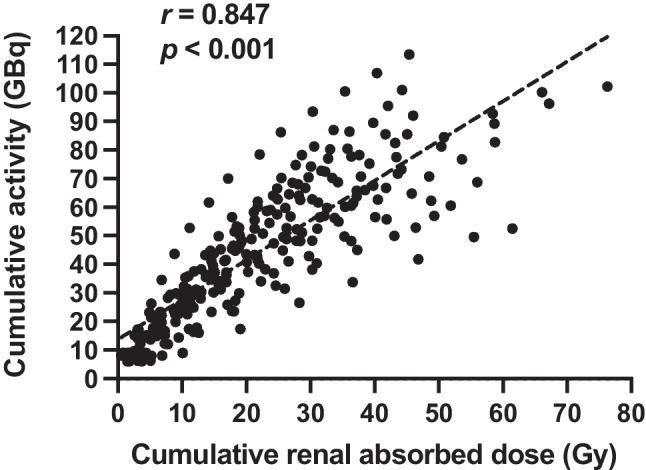
Cumulative renal-absorbed dose (Gy) after each cycle in relation to the cumulative activity after each cycle (GBq)

**Fig. 8:**
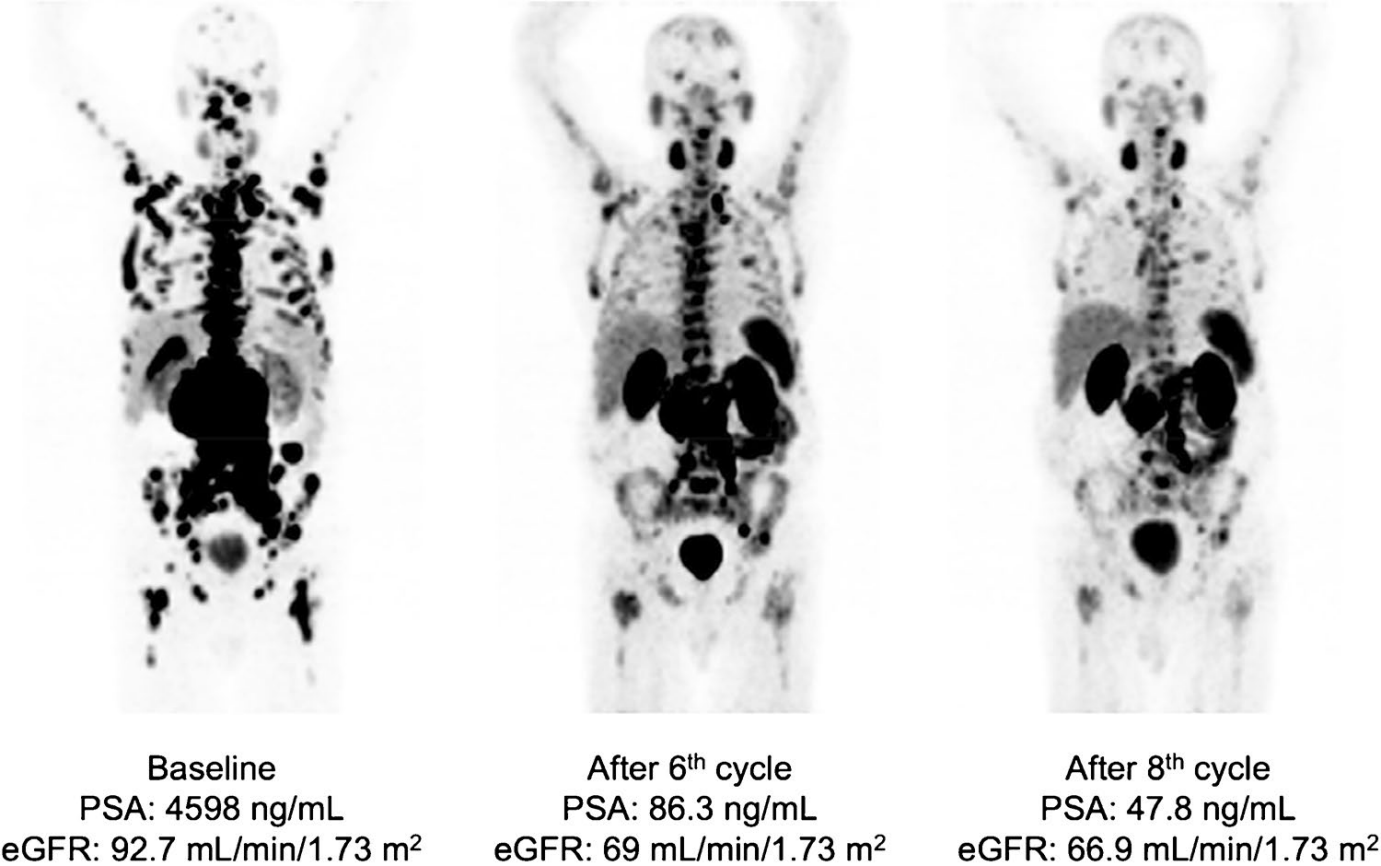
From left to right: MIP [^68^Ga]Ga-PSMA-11 PET images of a patient with high-volume disease receiving a low renal dose after initial cycles of [^177^Lu]Lu-PSMA RLT. Mean cumulative renal-absorbed dose was 14.5 Gy after a cumulative administered activity of 56.4 GBq, and Δ eGFR per year was − 13.8%. RLT was discontinued due to a cerebrovascular insult after 8 cycles

Mean administered activity was 7.4 ± 0.9 GBq per cycle, and mean renal absorbed dose was 0.54 ± 0.22 Gy/GBq resulting in a mean cumulative administered activity of 75.0 ± 19.1 GBq and absorbed total renal dose of 39.7 ± 15.3 Gy. Correlation of cumulative administered activity and cumulative absorbed renal dose up to each cycle is shown in Fig. 7 (*r* = 0.847, *p* < 0.001). Mean renal absorbed dose per administered activity increased from 0.47 ± 0.21 GBq/Gy during the first 6 cycles to 0.78 ± 0.13 Gy/GBq after additional cycles (*p* = 0.012) resulting in cumulative renal absorbed dose of 22.3 ± 10.3 Gy during standard treatment and 17.4 ± 11.3 Gy during additional 4 (IQR 2–6) cycles. A patient example with a cumulative administered activity of 56.4 GBq resulting in a cumulative absorbed renal dose of 14.5 Gy is presented in Fig. 8.

**Fig. 9:**
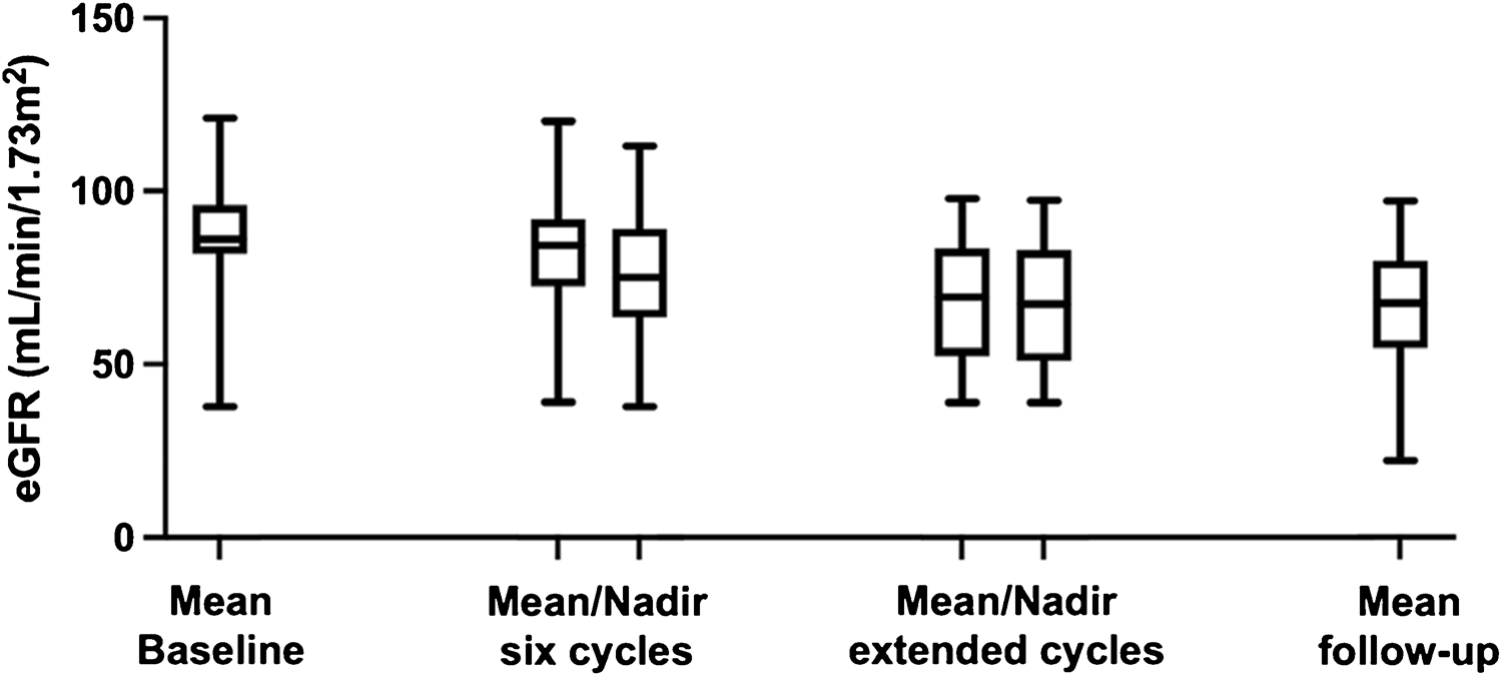
Mean eGFR at baseline, during the standard and extended [^177^Lu]Lu-PSMA RLT, and at the last follow-up

**Fig. 10:**
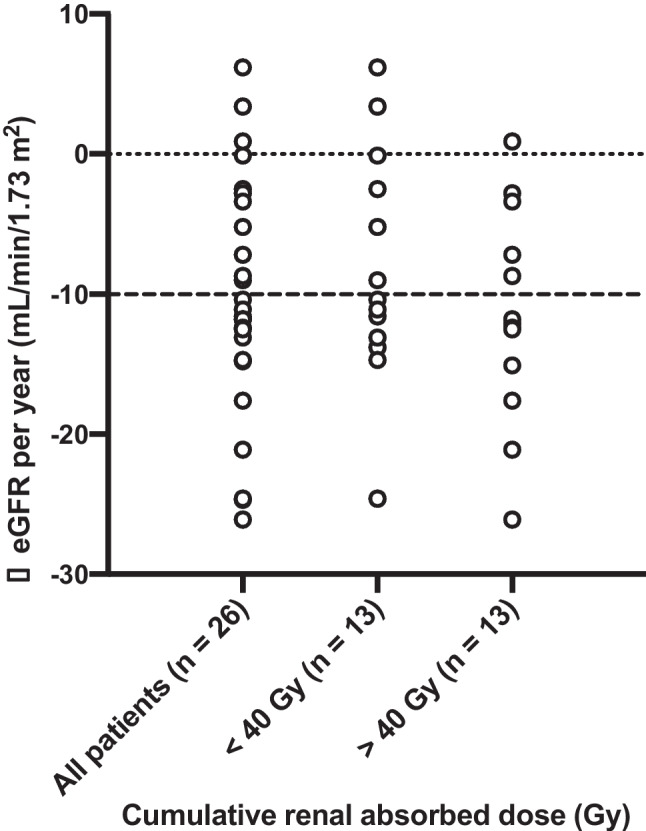
Yearly change in eGFR of all patients and separately in patients receiving cumulative absorbed renal doses < 40 Gy vs. > 40 Gy. The dashed line indicates a yearly change in eGFR of − 10 mL/min/1.73 m^2^

**Fig. 11:**
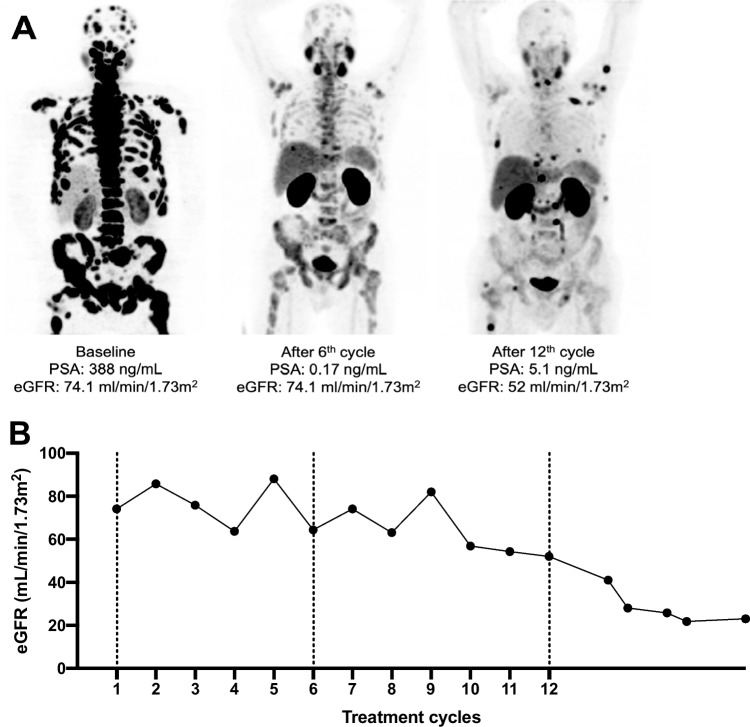
**A** From left to right: MIP [^68^Ga]Ga-PSMA-11 PET images of a patient with extensive disseminated bone metastasis and extradural infiltration. Extended RLT was discontinued upon progression after 12 cycles and a cumulative renal-absorbed dose of 63.3 Gy. **B** Course of eGFR from baseline to the last follow-up of the same patient, developing nephrotoxicity of grade 3

Mean eGFR levels changed from 85.3 ± 18.9 mL/min/1.73 m^2^ to 80.6 ± 16.7 mL/min/1.73m^2^ after the 6^th^ cycle (*p* = 0.025) and to 65.8 ± 19 mL/min/1.73 m^2^ after extended [^177^Lu]Lu-PSMA RLT at the last follow-up (*p* = 0.002; Fig. 9). Mean yearly change of eGFR in all patients was − 10.3 ± 8.5 mL/min/1.73m^2^/year. Cumulative absorbed renal dose of > 40 Gy was not significantly associated with a more prominent eGFR reduction per year (− 9 ± 9.5 vs. − 11.5 ± 7.5; *p* = 0.462) as shown in Fig. 10. Significant nephrotoxicity (grade ≥ 3) was observed in 1/26 (4%) patient 2 months after discontinuation of the extended [^177^Lu]Lu-PSMA RLT after having received 12 cycles (Fig. 11). No significant xerostomia was reported, but 6/26 (23%) patients developed xerostomia of grades 1 and 2 during the extended treatment and after a median of 10 (IQR 7–12) cycles. A summary of the adverse events is shown in Table 3.

**Table 3:** Hematologic, renal, and salivary gland toxicity grades based on CTCAE v5.0 at baseline, after 6 cycles, during added cycles, and at follow-up

Toxicity (grade)	Baseline (%)		After 6 cycles (%)		Added cycles (%)		Follow-up (%)	
	1/2	3/4	1/2	3/4	1/2	3/4	1/2	3/4
Anemia	15 (58)	0 (0)	26 (100)	0 (0)	23 (88)	3 (12)	22 (85)	4 (15)
Leukopenia	2 (8)	0 (0)	4 (15)	0 (0)	11 (42)	0 (0)	7 (27)	0 (0)
Thrombocytopenia	3 (12)	0 (0)	7 (27)	0 (0)	12 (46)	1 (4)	10 (38)	0 (0)
eGFR*	3 (12)	0 (0)	3 (12)	0 (0)	6 (23)	0 (0)	8 (31)	1 (4)
Xerostomia	0 (0)	0 (0)	9 (35)	0 (0)	15 (58)	0 (0)	15 (58)	0 (0)

^*^Estimated glomerular filtration rate

## Discussion

This retrospective study introduces the concept of extended [^177^Lu]Lu-PSMA RLT in mCRPC patients responding to [^177^Lu]Lu-PSMA RLT but completing standard treatment of six cycles with high residual tumor load. Patients received additional treatment cycles until disease progression, occurrence of significant toxicity, or achieving low PSMA-expressing tumor burden. Further tumor reduction in 38% (*n* = 10) of patients and a relatively long PFS of 19 months in our patients with high-volume disease and poor prognosis [12] indicate the clinical benefit of additional treatment cycles. Furthermore, the toxicity rate remained low in our patient group despite a cumulative administered activity of 75.0 ± 19.1 GBq, underlining the safety of [^177^Lu]Lu-PSMA RLT. These findings encourage extended treatment in selected patients with no other promising therapeutic options.

The current standard treatment scheme for [^177^Lu]Lu-PSMA RLT is based on previous experiences with peptide receptor radionuclide therapy (PRRT) for neuroendocrine tumors (NET) consisting of four cycles of 7.4 GBq [^177^Lu]Lu-DOTA-TATE per cycle [18]. This scheme was successfully adapted in the phase III VISION trial with a slight modification, and responders with residual tumor burden after the fourth cycle of [^177^Lu]Lu-PSMA-617 could be offered up to two extra cycles (i.e., six cycles) [6]. [^177^Lu]Lu-PSMA RLT with 1–6 cycles of [^177^Lu]Lu-PSMA-617 improved the survival of mCRPC patients as compared to standard of care, resulting in a PFS of 8.7 and an OS of 15.3 months [6]. No information was available regarding the number and outcome of the subgroup of responding patients receiving a total of six cycles. In our study, patients showed an OS of 29 months receiving extended [^177^Lu]Lu-PSMA RLT with a median of 10 cycles (range 7–16). This comparatively long overall survival may be partly due to the composition of our patient group consisting solely of responders with an expectedly more favorable outcome [9, 19]. The observed PFS of 19 months in our study is in line with the results of the only study addressing the outcome of patients receiving additional [^177^Lu]Lu-PSMA RLT cycles. In a study by Derlin et al. in 26 patients including 19 (73%) patients with a PSA decline ≥ 90% after six cycles, a median of 9 ± 2 cycles of [^177^Lu]Lu-PSMA-617, [^177^Lu]Lu-PSMA-I&T, or both was applied, aiming for a complete response (CR). Further PSA decline was seen in 13/26 (50%) patients. Two patients with biochemical CR showed imaging-based CR with no more PSMA-expressing lesions during the additional treatment period. A PFS of 15 months could be reached, but OS was not reported [8].

In patients with metastatic prostate cancer, high-volume disease according to CHAARTED (i.e., hepatic or multifocal bone involvement) is associated with poor prognosis [12]. This negative impact on survival has also been observed in the context of [^177^Lu]Lu-PSMA RLT. Ahmadzadehfar et al. observed a significantly shorter survival in patients with bone metastases (OS 10.8 months) undergoing [^177^Lu]Lu-PSMA RLT, and Khreish et al. reported an impaired outcome in the presence of hepatic metastases, even in the subgroup of patients responding to [^177^Lu]Lu-PSMA RLT with an OS of 14.5 months [20, 21]. Considerably longer overall survival of our patients despite high residual tumor load after six cycles indicates the beneficial effect of extended [^177^Lu]Lu-PSMA RLT.

[^177^Lu]Lu-PSMA RLT using the standard scheme has been proved safe with a low toxicity profile [6]. An increase in cumulative administered activity raises concerns regarding the safety of the treatment. Kidneys along with bone marrow are considered the main dose-limiting organs in patients undergoing [^177^Lu]Lu-PSMA RLT. In contrast to bone marrow suppression, treatment-induced renal function impairment may become clinically evident months after [^177^Lu]Lu-PSMA RLT initiation [22–26]. As a result, in patients with a life expectancy of more than 1 year, the cumulative kidney-absorbed dose may only exceed 40 Gy after careful individual benefit-to-risk evaluation [1]. Therefore, in our study, only patients with high total tumor volume and no other promising therapeutic option were selected for extended [^177^Lu]Lu-PSMA RLT. The mean cumulative kidney-absorbed dose was 39.7 ± 15.3 Gy, and 13/26 (50%) patients received > 40 Gy. Renal-absorbed dose per administered activity (0.54 ± 0.22 Gy/GBq) in our study is in line with the reported values in the literature, most probably due to the high tumor burden and evident tumor sink effect at baseline in our patients (Fig. 9) [1, 27]. Only 1/26 (3.8%) patient treated with a cumulated administered activity of 100.4 GBq resulting in a cumulative kidney-absorbed dose of 63.3 Gy developed significant kidney toxicity (grade 3) 18 months after the treatment initiation (Fig. 8). In the phase III VISION trial, grade 3 to 5 renal effects were observed in 3.4% after a median cumulative dose of 37.5 GBq (range, 7–48.3) and a follow-up of 20.9 months [6]. In the study by Derlin et al., no patient showed significant hematological or kidney toxicity (≥ grade 3) and only one patient discontinued [^177^Lu]Lu-PSMA RLT because of aggravating grade 2 nephrotoxicity; the cumulative kidney-absorbed dose was not reported [8]. In the phase III VISION trial, hematological adverse events (grade ≥ 3) including anemia, leukopenia, and thrombocytopenia occurred in 12.9%, 2.5%, and 7.9%, respectively [6]. In a retrospective study on 140 patients analyzing a total of 497 cycles of [^177^Lu]Lu-PSMA-617, significant (grade ≥ 3) hematologic adverse events occurred in 13 (9.3%) patients, with anemia in 10 (7.1%), leukopenia in 5 (3.6%), and thrombocytopenia in 6 (4.3%) [28]. Observation of significant hematological toxicity in 4/26 (15%) patients in our study supports the established favorable hematological safety profile of [^177^Lu]Lu-PSMA RLT even after high cumulative activities. Furthermore, the presence of progressive disease in 3 of these 4 patients suggested that significant toxicity may be at least partly attributed to tumor-induced bone marrow impairment [29].

There are some limitations in our study. The small sample size, hampering in-depth statistical analyses, and the retrospective nature, limiting the comparability with the prospective trials, need to be considered. However, improved survival outcome and low-toxicity rates in our patients justify a serious consideration of extended [^177^Lu]Lu-PSMA RLT in selected patients who respond to standard treatment but show high residual tumor load after the sixth cycle.

## Conclusions

Extended [^177^Lu]Lu-PSMA RLT may improve the survival outcome of mCRPC patients with high-volume residual tumor after the completion of standard treatment with six cycles of [^177^Lu]Lu-PSMA-617. Despite high cumulative activities, the safety profile may remain acceptable, warranting further investigation of the concept of extended treatment with additional cycles in selected patients.

## Data Availability

The datasets used and/or analyzed during the current study are available from the corresponding author on reasonable request.

## Abbreviations

ALP: Alkaline phosphatase
CI: Confidence interval
CTCAE: Common Terminology Criteria for Adverse Events
ECOG: Eastern Cooperative Oncology Group
eGFR: Estimated glomerular filtration rate
GBq: Gigabecquerel
Hb: Hemoglobin
IQR: Interquartile range
LDH: Lactate dehydrogenase
[^177^Lu]Lu-PSMA RLT: [^177^Lu]Lu-PSMA-617 radioligand therapy
mCRPC: Metastatic castration-resistant prostate cancer
NET: Neuroendocrine tumor
Plt: Platelets
PRRT: Peptide receptor radionuclide therapy
PSA: Prostate-specific antigen
PSMA: Prostate-specific membrane antigen
RLT: Radioligand therapy
VAS: Visual analog scale
WBC: White blood cells
